# From greening to browning: Catchment vegetation development and reduced S-deposition promote organic carbon load on decadal time scales in Nordic lakes

**DOI:** 10.1038/srep31944

**Published:** 2016-08-24

**Authors:** Anders G. Finstad, Tom Andersen, Søren Larsen, Koji Tominaga, Stefan Blumentrath, Heleen A. de Wit, Hans Tømmervik, Dag Olav Hessen

**Affiliations:** 1 Department of Natural History, NTNU University Museum, Norwegian University of Science and Technology, N-7491 Trondheim, Norway; 2 Norwegian Institute for Nature Research, P.O. Box 5685 Sluppen, NO-7485 Trondheim, Norway; 3 University of Oslo, Department of Biosciences, P.O. Box 1066, Blindern, 0316 Oslo, Norway; 4 Norwegian Institute for Water Research, Gaustadalléen 21, NO-0349 Oslo, Norway

## Abstract

Increased concentrations of dissolved organic carbon (DOC), often labelled “browning”, is a current trend in northern, particularly boreal, freshwaters. The browning has been attributed to the recent reduction in sulphate (S) deposition during the last 2 to 3 decades. Over the last century, climate and land use change have also caused an increasing trend in vegetation cover (“greening”), and this terrestrially fixed carbon represents another potential source for export of organic carbon to lakes and rivers. The impact of this greening on the observed browning of lakes and rivers on decadal time scales remains poorly investigated, however. Here, we explore time-series both on water chemistry and catchment vegetation cover (using NDVI as proxy) from 70 Norwegian lakes and catchments over a 30-year period. We show that the increase in terrestrial vegetation as well as temperature and runoff significantly adds to the reduced SO_4_-deposition as a driver of freshwater DOC concentration. Over extended periods (centuries), climate mediated changes in vegetation cover may cause major browning of northern surface waters, with severe impact on ecosystem productivity and functioning.

The observed increased concentrations of dissolved organic carbon (DOC) of northern boreal lakes during the past two to three decades, often termed “browning”^1^,^2^ has attracted considerable attention due to the role of DOC in the global carbon cycle^3^, as well as its multitude of impacts on ecosystem processes and services^4^,^5^,^6^.

The browning has been attributed to various ambient drivers related to sulphate (SO_4_^2−^) deposition, climate, hydrology and land-use^1^,^7^,^8^. Reduced sulphate deposition affects the export of DOC from terrestrial pools into freshwaters by altering the solubility of organic matter^1^,^7^,^8^. The underlying mechanisms have been suggested to involve a combination of increased charge density of humic substances and reduced ionic strength, both contributing to increasing organic matter solubility^7^,^8^. Also increased temperature and altered hydrology, enhancing soil carbon decomposition rates and the amount of water flow through soil as well as retention time, respectively, have been suggested to be important drivers of changes in DOC-fluxes^9^,^10^. The main source of coloured, allochthonous DOC in boreal surface water is terrestrially fixed organic carbon^3^ with origin from forests and peatlands^11^,^12^, and increases in soil organic matter pools, mediated by increases in terrestrial productivity, have been documented to promote DOC in surface waters on long (century) time scales^13^.

Currently, there is a prominent “greening” trend in many northern and alpine areas, i.e. increased vegetation, biomass carbon pools and/or forest cover. This trend has been attributed to climate warming, nitrogen deposition, reduced grazing activities and reduced forestry harvesting intensity^14^,^15^. Forest inventories and remote sensing studies have revealed a large increase in standing forest mass of Northern temperate areas over the past decades^16^. Also, a pronounced increase in growing season has been recorded over northern latitudes^17^. In a survey of 1000 Norwegian lakes and catchments, vegetation cover represented by the remote sensing derived Normalized Difference Vegetation Index (NDVI) was found to be the key spatial predictor of DOC, followed by bogs^18^. Linked to space-for-time predictions of changes in NDVI under a 2 °C climate scenario, this approach was used to predict a profound future browning of northern waters^19^. A large portion of riverine DOC export is constituted by “modern” (decade-old) carbon^20^,^21^ suggesting that an increase in terrestrial vegetation biomass and productivity in the course of decades may contribute to increases in DOC. However, empirical support for terrestrial carbon fixation promoting browning in boreal surface waters on a decadal timescale has yet not been presented.

In the present study, we investigate to what extent the observed greening of terrestrial vegetation contributes to changes in surface water DOC. We used time-series (1986–2013) of measured DOC from annual water samples in 70 lakes, and extracted annual composites of catchment vegetation proxies along with along with annual composites of the other suggested main drivers of the observed increase in DOC: catchment sulphur (S) deposition, temperature and runoff. The study sites were all from Norway and cover primarily pristine boreal headwater catchments along a large climatic gradient, as well as a gradient of sulphate deposition. All lakes in this region have experienced a substantial decline in S deposition since the 1980s^2^ resulting in considerable post-acidification chemical recovery.

## Results

**Figure 1:**
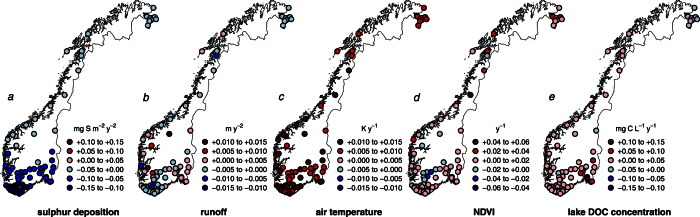
Lake specific trends in total atmospheric S deposition, runoff, surface air temperature, NDVI and lake DOC concentration. Estimated Theil-Sen’s slope values (y^−1^) based on site-specific Regional Kendall Tests for (**a**) catchment total atmospheric S deposition, (**b**) runoff, (**c**) surface air temperature, (**d**) NDVI and (**e**) lake DOC concentration during the study period (1986–2011). Positive temporal trends are depicted using shades of red, whereas negative temporal trends are depicted using shades of blue. A stronger shade in either trend direction denotes a faster trend when compared regionally. The categorization levels were determined using R’s *pretty* function on absolute slope values^73^. All S deposition trends where negative, and all temperature trends where positive, 85% of the DOC trends and 76% of the NDVI trends were positive, and 65% of the catchments displayed negative runoff trends. Overall, the mean regional trends for the whole study area for all five variables were significant (positive for NDVI, temperature and DOC, negative for S deposition and runoff, Regional Kendall Tests with lakeID as block, *n* = 70, all with *p* < 0.017). Figure created in R v. 3.2.1 (URL http://www.R-project.org/)^61^ using the libraries raster^60^ and sp^74^.

There was an increasing trend of DOC in the majority of the lakes during the study period (1986–2013) (Fig. 1) which corresponded to an average decadal increase of 1.7 mg l^−1^. The strongest increase in DOC was observed in the most acidified areas in south-eastern Norway which also have seen the strongest decreases in S-deposition. This is consistent with previously reported increase of organic carbon in many European and North American freshwaters^2^,^22^, and supports the strong link between decreased S deposition and increased DOC in previously acidified areas^1^.

**Figure 2:**
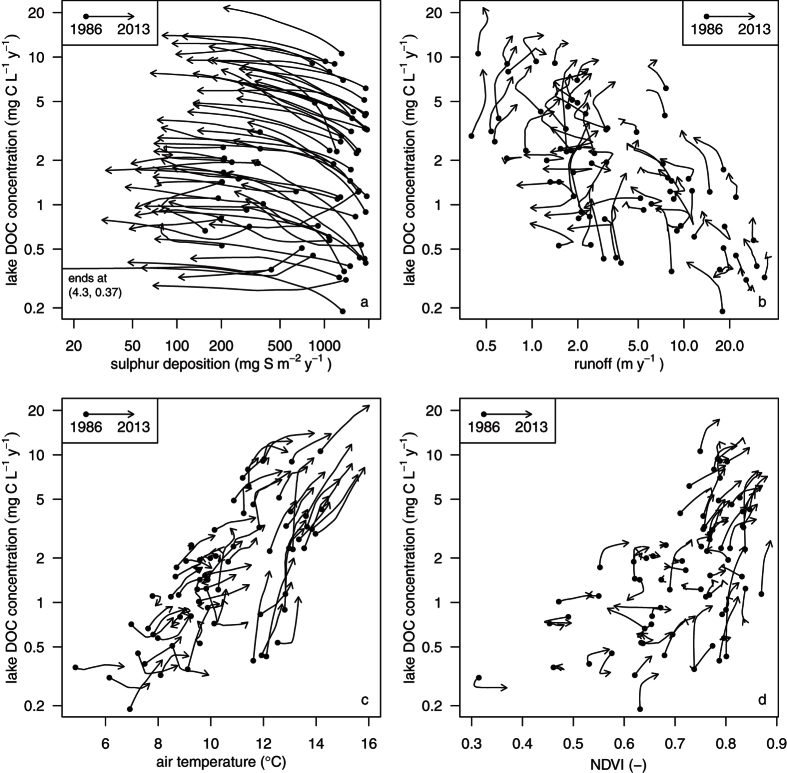
Pairwise time-trend relations between individual driver variables and DOC. Two-dimensional cross smoothed time series curves starting from 1986 (dots) to 2013 (arrow heads) for individual lakes, where DOC concentration is plotted against (**a**) sulphur deposition, (**b**) runoff, (**c**) surface air temperature and (**d**) NDVI. Each curve represents a time series for a lake. Coordinates were obtained by smoothing lake-specific time series using lowess^61^ with a smooth span of 0.8. Only those years with a complete pair of observations are used in this figure. Lake DOC concentration, total sulphur deposition and runoff are scaled logarithmically.

Catchment vegetation (NDVI) and temperature also showed increasing trends (Fig. 1) 76% of the catchments had a positive NDVI trend, with a mean decadal increase of 5.2%. This supports previous findings of an increase in vegetation coverage or density in many northern and boreal areas^14^,^15^,^23^. Some areas had no response, and high altitude sites (above ca. 1000 m) in southern Norway showed a decreased trend for NDVI. The negative NDVI-trends in truly alpine areas possibly reflects increased precipitation as snowfall, hence later snowmelt and decreased growing season^17^,^24^, but could also be attributed to vegetation damage due to extreme weather events, herbivory and pests^25^. Runoff in the majority of the catchments tended to decline, but with no clear regional pattern (Fig. 1). We additionally regressed the trend slopes of DOC against the trend slopes of the investigated potential driver variables (Tables S10 and S11) using a multiple linear regression. Comparison of standarized model coefficients showed that the strongest contribution to the observed increase in DOC came from the reduction in S deposition (−0.24), followed by runoff (−0.19) and temperature (0.15) and NDVI (0.08). For a more direct visualization of trends we plotted pairwise smoothed time series curves for the relationship between lake DOC and S deposition, runoff, temperature and NDVI (Fig. 2).

Further, we used DOC and annual means for NDVI, S-deposition, runoff and temperature in a mixed effect multiple regression model with lake as a random factor, and a temporal trend (year) effect nested within lake as a controlling variable to account for time trends that cannot be represented by the explanatory variables in our models. There was strong support for the effect of S deposition, catchment vegetation (NDVI), but also runoff (Table 1). Models with different time lags gave similar relative importance rankings for the explanatory variables. Despite the robustness of the general model predictions we were unable to produce one single best model. Accordingly, model averaging was used for summarizing coefficients (Table 2). Judged from the standardized coefficients and the relative influence of the selected variables (RI), the most important explanatory variables were S deposition (1), followed by runoff (0.96) and NDVI (0.93). There was a smaller, but still significant, effect of temperature (0.26) (Table 2). The pronounced contribution from changes in catchment vegetation suggests that increased terrestrial C-fixation promotes export of allochthonous C to surface waters on a decadal timescale. This finding complements previous studies that demonstrated a strong spatial effect of NDVI on DOC^18^, and which was used to predict an increase in DOC as a result of a climate-induced landscape greening^19^.

## Discussion

Our analysis agrees with the previously documented effect of reduced S deposition (or lake S-concentration) on DOC^1^, and also supports the role of NDVI as a proxy of terrestrial vegetation promoting aquatic DOC^19^. This is, however, the first study to suggest that terrestrial carbon fixation indeed promotes browning in boreal surface waters on a decadal timescale. The role of coniferous forests along with peatlands as a major source of DOC has been confirmed for boreal catchments in general^11^,^12^,^26^. Thus, it seems reasonable to assume that increased terrestrial primary production or forest cover will induce an increase in DOC export. Since S-deposition is now approaching pre-industrial levels and thus is unlikely to decrease much further, and because terrestrial greening may continue for centuries to come, it can be assumed that the relative impact of vegetation on aquatic DOC will increase.

Climate change has been shown to increase vegetation cover and plant biomass (greening) of northern latitude landmasses during the past few decades^17^. For example, in Norway, tree-lines have moved upwards by more than 40 meters or advanced northwards by 15 km, likely as a combined response of climate change, reduced grazing by domestic animals and elevated nitrogen deposition^27^,^28^,^29^. Also, forest biomass has increased with 27% since 1990 15, comparable with biomass changes in Sweden^30^,^31^ and Finland^30^. The increase in biomass has in part been attributed to reduced harvesting intensity in managed European forest but climate warming and N deposition^32^ are also promoting forest growth. The tree-line advancement and greening of Arctic and boreal regions may not be a uniform response, however, as there may be both spatial and temporal differences^29^,^33^,^34^. In fact there may be regions also experiencing temporal set-backs of terrestrial vegetation. More than 5% of the vegetation in the boreal region and 4% of the Arctic region have experienced periods of “browning” in the period 1981–2011 17. Recent observations shows that the northern part of Fennoscandia have experienced winter warming events that caused detrimental damage to vegetation^25^,^35^ and caterpillar attacks on mountain birch forests^36^. The short and long terms impacts of such browning events of catchment export of DOC are not settled, however.

Feedbacks from declining snow-cover extent leading to longer growing seasons are promoting vegetation compositional/structural changes. Enhanced nitrogen mineralization in warmer soils insulated by increased shrub cover^17^,^37^ may also contribute to the elevated DOC. For taiga and tundra areas, increased permafrost thaw and changing vegetation cover over extended timescales also promote elevated DOC-levels of surface waters^38^,^39^,^40^. The catchments included in our study do not have permafrost, with potential exception for the northernmost areas where DOC changes are relatively small. For catchments at higher elevation is has been suggested, however, that permafrost declines during the last decades may have locally increased the export of organic carbon, as well as sulphur and nutrients^39^,^41^,^42^.

Changes in redox conditions mediated by combinations of hydrology and iron (Fe) reduction and oxidation cycles have been proposed as drivers of elevated DOC^43^,^44^. Fe may play a twofold role in this context, both as a mobilizer of DOC and SO_4_ itself^44^, and also by its chromophoric properties, making the DOC more “coloured”^43^, thus attenuating more light^45^. Also, we may expect increases in DOC of surface waters due to changing temperature and precipitation patterns, as well as associated changes in bogs and wetland.

The impacts of freshwater browning are manifold, e.g. reduced drinking water quality, changes in food web structure and productivity^5^,^46^, as well as increased anoxia^47^,^48^. Increased levels of coloured, allochthonous DOC will affect aquatic productivity at various trophic levels in numerous ways. Organic carbon may stimulate heterotrophic productivity from bacteria^49^ to fish^50^, or indirectly promote autotroph production via elevated levels of CO_2_ 51, increased attenuation of detrimental UV-radiation^52^ or increase net heterotrophy through enhanced microbial oxygen consumption of bioavailable DOC^47^. Also nutrients associated with dissolved organic material may stimulate autotroph productivity in nutrient-poor areas^5^. However, the overall net effect of increased DOC on aquatic productivity is likely negative. This is primarily attributed to its shading effect, with increased light attenuation causing reduced primary production^45^,^53^, as well as promoting benthic anoxia^48^,^54^. This may cause negative impacts up the trophic ladder to fish which will suffer both from bottom-up cascade effects as well as reduced search field for prey due to reduced visibility^5^,^55^. Downstream effects of increased terrestrial export of DOC and sediments have been observed in recipient coastal areas^56^, with corresponding negative impacts on marine primary and secondary production, and potentially a shift from visual predators (fish) to non-visuals (gelatinous plankton).

The wide range of potential impacts on productivity and community composition thus calls for more knowledge of short- and long-term (i.e., inter-annual and decadal to centuries) drivers of browning, which again can serve as basis for future predictions of impacts on aquatic systems.

## Methods

### Lake chemistry data

The data in this analysis are obtained from a Norwegian lake monitoring program where 70 lakes covering the Norway mainland have been sampled annually from 1986 to 2013 (Fig. 1). These lakes represent acid sensitive, headwater lakes on granitic or gneissic bedrock with negligible local pollution sources. Water samples were collected at the outlet after the autumn circulation period and analysed at the Norwegian Institute of Water Research^57^. Due to that the analytical protocol measures organic carbon on unfiltered water, we use total organic C (TOC) as a proxy for DOC. In these boreal, low-productivity systems TOC are for this purpose substitutable with dissolved organic carbon (DOC) since DOC typically constitutes >90% of TOC^58^.

#### Environmental drivers

Lake catchments where delineated from a 10 m digital terrain model (obtained from The Norwegian Mapping Authority)^59^. Time series of annual mean catchment vegetation biomass/productivity (NDVI), runoff, temperature and sulphur deposition were then extracted using the raster library^60^ in R v. 3.2.1^61^.

We used NDVI (Normalized Difference Vegetation Index; GIMMS NDVI3g) as proxy for catchment vegetation biomass/productivity^62^. Although care should be taken before interpreting NDVI changes directly as net changes in primary production, the index has previously proven a good surrogate of photosynthetic activity and for capturing long-term changes in vegetation^63^. The NDVI3g data set has a pixel resolution of 8 × 8 km and is recorded as bimonthly maximum NDVI. Here, we used the mean NDVI3g record for the main snow free period (June–August) averaged annually from each catchment area. Other composites, such as maximum yearly NDVI3g and June–August maxima gave similar results in terms of temporal dynamics, and were therefore not included in further analyses.

Time series of temperature and runoff for individual catchment were extracted using gridded data from 1980 to 2013 (1 km^2^ resolution from the Norwegian Meteorological Institute and the Norwegian Water Resources and Energy Directorate^64^. The original data with a daily resolution were aggregated to June to August means for individual catchments, as for NDVI time series.

Since S concentration in lake water may originate from various sources and also be confounded by hydrology and temperature, we here use time series of S deposition at the catchment level which were interpolated from EMEP deposition raster temporal composite (1980, 1985, 1990, and annually from 1995 to 2011)^65^ using recalculations if available. Total S deposition was used in the present study, comprising both dry and wet deposition. Each type of deposition was modelled using regression kriging using EMEP precipitation as a linear regression model predictor, with residuals fitted using a linear variogram model with no nugget. Using the variogram model and linear model, deposition flux over the catchment area was predicted using block kriging with the target geometry being the catchment polygon. Missing values from 1985–1990 and 1990–1995 where estimated within each catchment using loess regression^61^. For comparison we did also run the model with SO_4_ concentrations measured as part of the monitoring program, which yielded the same basic predictions as for deposition data (see Table S8 and S9).

#### Data standardization and software

All pre-processing of data and analyses was done in R v. 3.2.1^61^. Prior to the analyses, DOC were log transformed, and all variables standardized by subtracting mean and dividing on standard deviation.

#### Analyses

We first extracted temporal trends (Theil-Sen’s slopes)^66^ for lake DOC and catchment specific NDVI, run-off, atmospheric S deposition using the rkt library^67^. We tested for overall trends in DOC and each of the catchment drivers using a regional Kendall trend test from the same library, using the individual lake as block^66^. Additionally, we illustrated the concordance between the temporal trends by using the Theil-Sen slopes for DOC as dependent variable and corresponding slopes for catchment specific NDVI, run-off, and atmospheric S deposition as explanatory variables in a generalized least-squares regression model (gls). We compared models with and without spatial autocorrelation structure (fitted by REML) using Akaike’s information criterion (AIC) (Zuur *et al*. 2009). However, no support for inclusion of a spatial autocorrelation was apparent (∆AIC > 4.00 in support of model without spatial autocorrelation). Although such analyses have some issues in terms of using Theil-Sen slopes as input variables by for example disregarding the inherent between-year variation in vegetation measures, they nevertheless provide a useful illustration. See supplementary material (section D) for further description.

We then included effects of S deposition, catchment NDVI, runoff, and air temperature as driver variables in a linear mixed effect model with DOC as dependent variable (*nlme* library)^68^. Year was initially included in the full model as a controlling variable^69^ in order to control for potential temporal trends in DOC not accounted for by our explanatory variables. Model selection and parameter estimation re-runs with and without year as controlling variable did not indicate that this had any major effects on the main conclusions (see supplementary material Table S4 and S5). Selection of random structure where done on a full fixed model structure (fitted by REML) by model comparison^70^. This resulted in a full model including lake-ID as random intercept and year nested within lake-ID as random slope (∆AIC > −76.65 in favor of the selected random structure). By introducing lake ID as random factor, we model between-lake variation in DOC caused by catchment variables not considered in the current study (static land cover properties that not are expected to change on the relevant time span such as bogs and wetlands, catchment size, lake size, etc.). Temporal autocorrelation (AR1) was initially also tried, by entering year nested within lake-ID, but proved redundant in the initial selection of the random structure. There was no signs of multi-collinearity (variance inflation factors^70^, all VIF < 1.96, and maximum correlation between predictors where 0.61, see Fig. S2).

Catchment vegetation may be expected to influence lake DOC with certain time lags. However, in order to avoid data-dredging issues, we selected a-priori a one year’s time lag for NDVI and runoff as the input for our analyses, but subsequently performed sensitivity analyses on the effect of the time lag chosen (see Figure S4). The main predictions were insensitive to choice of time lag.

#### Model selection and multi-model inference

Model selection and model averaging on fixed effect structure where done by model comparison using the MuMIn library^71^. All possible combinations of fixed factors where compared and candidate models ranked according to the Akaike information criterion (AIC). Since there was no clear top-ranked candidate model, we applied Akaike weight-based averaging over the 95% confidence model set (cumulative AIC weights of models ≥0.95) to estimating coefficients for the candidate models as well as their 95% confidence intervals. The relative influence (RI) of each variable was given as the summation of *w*_*i*_ across all models including that variable in the 95% confidence model set^72^.

Residuals of the final selected models were visually inspected for deviations from normality, heteroscedasticity, and spatial or temporal autocorrelation without finding evidence for violation of model assumptions (see Supplementary material).

## Additional Information

**How to cite this article**: Finstad, A. G. *et al*. From greening to browning: Catchment vegetation development and reduced S-deposition promote organic carbon load on decadal time scales in Nordic lakes. *Sci. Rep*. **6**, 31944; doi: 10.1038/srep31944 (2016).

## Supplementary Material

Supplementary Information

## Figures and Tables

**Table 1:** Model selection tables for the mixed effect model of mean yearly total organic carbon (DOC) against normalized difference vegetation index (NDVI), runoff (Q), Sulphur deposition (S-dep), temperature (T), as well as year.

Intercept	NDVI	Q	S-dep	T	year	n	logLik	AIC	∆i	Wi
−0.150	0.046	−0.056	−0.098		0.148	9	−189.69	397.4	0.00	0.63
−0.151	0.046	−0.058	−0.098	−0.008	0.150	10	−189.56	399.1	1.72	0.27
−0.147		−0.068	−0.103		0.143	8	−192.94	401.9	4.50	0.07
−0.153	0.057		−0.101		0.150	8	−193.39	402.8	5.38	0.04

The tables show parameter estimates for model terms included in the models, number of model parameters (n), log likelihood (LogLik), AIC, AIC difference from best model (∆i), and Akaike weights (Wi). Only models from the top 95% confidence model set shown (cumulative AIC weight of models ≥ 0.95).

**Table 2:** Summary result for model averaging of fixed effects in the 95% confidence model set (cumulative *W*_*i*_ ≥ 0.95) on total organic carbon (DOC) against normalized difference vegetation index (NDVI), runoff (Q), Sulphur deposition (S-dep), temperature (T), as well as year.

	Estimate	SE	95% CI	Relative importance
Intercept	−0.151	0.105	(−0.356, 0.055)	
NDVI	0.047	0.018	(0.010, −0.082)	0.93
Q	−0.057	0.021	(−0.098, −0.016)	0.96
S-dep	−0.098	0.023	(−0.144, −0.052)	1
year	0.147	0.038	(0.073, 0.223)	1
T	−0.008	0.015	(−0.038, 0.022)	0.26

Parameter estimates (on standardized scale) are interpretable as effect size (i.e. describes changes in unites of standard deviation of the original variable), unconditional SE, 95% Confidence intervals and relative importance.
