# Supplementary material for: From greening to browning: Catchment vegetation development and reduced S-deposition promote organic carbon load on decadal time scales in Nordic lakes
Source: Sci Rep. 2016 Aug 24;6:31944. doi: 10.1038/srep31944 (PMC4995398; doi:10.1038/srep31944)
Supplement: Supplementary Information [file srep31944-s1.pdf]

Supplementary information to 'From greening to browning: Catchment vegetation development and reduced S-deposition promote organic carbon load on decadal time scales in Nordic lakes'

Anders G. Finstad, Tom Andersen, Søren Larsen, Koji Tominaga, Stefan Blumentrath, Heelen A. de Wit, Hans Tømmervik, Dag Olav Hessen

---

Supplementary material contains contains four parts:

A) Notes on methodological differences compared to pre-sequel studies

B) Additional sampling site and source data description

C) Extended output and additional analyses, including residual plots for the mixed effect modelling

D) Additional analyses and residual plots for supplementary trend analyse (using regression slopes as modell inputt for correspondence with previous studies)

A) Notes on methodological differences compared to pre-sequel studies

The current study builds upon previous studies demonstrating increases in DOC of boreal lakes and rivers (de Wit et al. 2007, Monteith et al. 2007). These studies have used concentrations of SO4 analyzed from water same samples as proxy for Sulphur deposition. Here, we use atmospheric Sulphur deposition directly as input variable. The rationale for this is that SO4 in water partly is controlled by the same drivers as DOC (hydrology not the least), which could induce spurious correlations. Climate, and notably hydrology, will affect oxidation and mobilization of Sulphur in soils and bogs that also contribute to SO4 in recipient waters (Hongve et al. 2004), implying that there, at least on annual scales, will be deviations between input and output of SO4 from catchments. Deposition of SO4 is, however, calculated by interpolating data from a number of monitoring stations (see below), while SO4 in water is measured with high accuracy. Secondly, previous studies are mainly based upon correlations between observed trend metrics (slopes) of DOC and driver variables over time. We here apply a regression-based model with yearly mean values as input. Additionally, we used ln DOC as dependent variable (hereafter termed ln TOC in the supplementary, see material and methods in main text), where the anti-log of

the regression coefficients can be interpreted as relative change from one year to the next. Utilizing individual data-points furthermore increase the statistical power of the analyses and enables inspections of time-lags. We provide a replication of the analyses using trend metrics (slopes) in supplementary material, section D). Although trend metrics still provide a significant effect of vegetation, the relative importance of vegetation is here less due to the inherent between year variation climate variation induce in vegetation measures. The long term trends of climate on vegetation proxies hence become weaker than the relatively linear decrease in Sulphur deposition experienced throughout the last decades.

B) Additional sampling site and source data description

Lake chemistry data

The data in this analysis are obtained from a Norwegian lake monitoring program from 70 lakes covering the Norway mainland and with annual samples from 1986 to 2013. These lakes constitutes a subset of the 1000-lake acidification survey of 1995 (Henriksen et al. 1998, Ambio 27:80-91), and represents acid sensitive, headwater lakes on granitic or gneissic bedrock with negligible local pollution sources. Water samples are collected annually at the outlet after the autumn circulation period and where analysed at the Norwegian Institute of Water Research (Skjelkvåle et al. 2001,Hydrol. Earth Syst. Sci. 5:327-338). The original dataset (available from URL http://vannmiljo.miljodirektoratet.no/)contains information from 78 lakes, but eight lakes where excluded from the present study due to incomplete catchment data. Catchments crossing national borders did not have complete data on runoff (provided by The Norwegian Water Resources and Energy Directorate).

Table S1: List of study sites (lakes) included in the present study. The table is given with lakeID corresponding to the official lake numbering given by the Norwegian Water Resources and Energy Directorate, http://www.nve.no/en/, latitude and longitude (EPSG:4326) of lake centroid, lake area (square kilometers), catchment area (square kilometres) and mean lake TOC concentration.

LakeID	Latitude	Longitude	lakeArea	catchmentArea	TOC
69	59.83574	8.737854	1.82	10.33	0.81
282	61.15767	11.627560	1.14	5.67	6.78
331	59.10063	11.522082	1.15	7.91	5.43
368	60.09764	11.902080	1.16	34.97	11.22
716	65.06711	13.168821	2.78	10.93	1.14
806	66.75356	15.410483	2.60	46.24	0.73
1001	68.08597	16.045089	1.41	6.29	2.29
1094	59.58272	7.563858	1.15	7.50	0.34
1174	58.74887	7.308764	0.96	5.53	2.60

1373	58.63211	6.976235	0.27	11.50	5.59
1431	58.48877	6.735423	1.20	6.51	1.03
1545	58.56277	5.867287	0.53	8.08	1.06
1651	61.34421	6.493807	1.27	22.51	0.38
1935	61.98776	6.183566	1.04	21.18	1.38
2430	70.34170	30.859106	2.74	8.08	1.14
2437	69.94011	29.118845	1.79	7.40	0.78
2456	69.27195	28.954739	2.60	8.76	2.06
2474	69.69150	30.667201	3.59	19.82	1.17
3238	59.77423	11.762489	0.52	2.95	3.67
3555	59.11542	11.677879	0.24	2.12	8.15
3838	60.55665	11.665238	0.46	20.99	12.49
5114	60.10521	10.757073	0.55	7.85	3.53
5269	59.97221	10.145206	0.31	1.44	6.03
5742	59.63713	10.103627	0.36	5.22	7.59
5828	59.40272	11.001102	0.29	3.08	4.89
5844	59.34441	10.969082	0.20	3.28	16.68
5961	59.89208	9.305583	0.08	4.67	10.66
7272	60.36662	9.730464	0.23	4.55	11.46
9219	58.74638	7.954483	0.33	8.84	3.94
9534	58.69217	8.963088	0.22	3.70	7.00
10127	58.59644	8.544524	0.31	2.28	4.12
10305	58.54989	7.204447	0.26	3.32	8.20
10926	58.38866	7.973851	0.29	3.81	4.42
11078	58.31903	7.679679	0.24	13.03	7.44
11095	58.30425	7.158552	0.34	10.37	5.21
11147	58.29347	7.924448	0.22	10.80	5.68
11292	58.22903	6.992603	0.23	1.06	2.16
11373	58.20758	7.452243	0.75	3.81	2.44
11592	58.11910	7.665664	0.45	18.69	4.99
13194	59.62932	8.116062	0.41	2.26	1.49
13592	59.49601	7.114335	1.50	16.54	0.26
14277	59.29748	7.715758	1.27	4.01	0.86
14367	59.27227	8.843790	0.11	4.95	8.84
14534	59.20990	7.242493	0.69	9.22	0.50
15100	59.07598	7.445520	0.70	5.59	2.23

15177	59.05965	7.378440	0.61	33.68	2.43
21049	58.52418	6.419590	0.38	2.07	1.07
21186	58.48700	6.188944	0.36	1.51	0.80
21438	58.41618	6.212963	0.29	1.74	0.68
21797	58.28628	6.493815	0.67	12.41	1.29
22101	59.87227	5.430483	0.26	4.62	2.32
22548	59.54436	6.024239	0.43	16.94	1.42
23386	59.81531	6.353889	1.09	26.72	0.45
26267	60.72892	5.505261	0.43	2.89	0.67
28197	61.67410	5.164808	0.70	2.76	0.74
31047	62.04337	5.786375	0.56	1.90	0.35
31186	62.82214	7.522910	0.31	4.82	3.43
34660	62.27806	8.843371	0.60	50.15	0.34
35326	62.60575	11.897614	1.37	4.94	1.91
36436	63.29230	8.770949	0.50	7.95	1.64
40844	64.28040	10.974211	1.01	3.61	2.90
45724	67.75659	15.958838	1.07	8.75	1.84
48048	68.05404	13.326508	1.20	6.37	0.95
50879	69.24335	17.316576	0.70	18.07	0.89
63664	69.86779	29.185201	0.69	2.53	0.90
64217	69.70881	30.601720	0.41	1.76	0.63
64278	69.70551	29.746762	0.97	2.94	1.57
64684	69.57107	29.813285	0.45	6.90	2.11
64713	69.55057	30.779834	0.18	6.75	2.39
64799	69.53605	29.459653	0.22	3.32	1.75

Environmental drivers

Lake catchments where delineated from a 10m digital terrain model (available from The Norwegian Mapping Authority). Time series of annual mean catchment vegetation biomass/productivity (NDVI), runoff, temperature and sulphur deposition where then extracted using the raster library (*Hijmans 2015, raster: Geographic Data Analysis and Modeling. R package version 2.4-15*) in R v. 3.2.1 (*R Core Team 2015, R: A language and environment for statistical computing. R Foundation for Statistical Computing, Vienna, Austria. URL http://www.R-project.org/*).

We used NDVI (Normalized Difference Vegetation Index; GIMMS NDVI3g) as proxy for catchment vegetation biomass/productivity (*Fensholt and Proud 2012, Remote Sensing of Environment 119:131-147; Pinzon and Tucker 2014, Remote Sensing*

6:6929-6960). The NDVI is a radiometric measurement of the fraction of photosynthetically active radiation (~ 400 to 700 nm) absorbed by chlorophyll in the leaves of a vegetation canopy (*Myneni et al. 1995, Trans. Geosci. Rem. Sens. 33:481-486*). Although care should be taken before interpreting NDVI changes directly as net changes in primary production, the index has previously proven a good surrogate of photosynthetic activity and for capturing long-term changes in vegetation (*Myneni et al. 1997, Nature 386:698-702; Forkel et al. 2013, Remote Sensing 5:2113-2144*). The NDVI3g data set has a pixel resolution of 8 x 8 km. The maximum NDVI value over a 15-day period is used to represent each 15-day interval to minimize bias due to cloud contamination and variations in atmospheric turbidity, scan angle, and solar zenith angle (*Holben 1986, Int. J. Rem. Sens. 7:1417-1434; Pinzon and Tucker 2014, Remote Sensing 6:6929-6960*). This scheme results in two maximum-value NDVI composites per month. Here, we used the mean NDVI3g record for the main snow free period (June - August) averaged annually from 1982 to 2011 and over each catchment area. Other measurements, such as maximum yearly NDVI3g and June - August maxima gave similar results in terms of temporal dynamics, and were therefore not included in further analyses.

Time series of temperature and runoff for individual catchment were extracted using gridded data from 1980 to 2013 (1 km2 resolution from the Norwegian Meteorological Institute and the Norwegian Water Resources and Energy Directorate (*Mohr 2008, New Routines for Gridding of Temperature and Precipitation Observations for http://www.seNorge.no*). The original data with a daily resolution were aggregated to June to August means for individual catchments, corresponding to the NDVI time series.

Time series of S deposition at the catchment level was interpolated from EMEP deposition raster temporal composite (1980, 1985, 1990, and annually from 1995 to 2011) (*Schulz et al. 2013, Transboundary acidification, eutrophication and ground level ozone in Europe in 2011. Norwegian Meteorological Institute*) using recalculations if available. Total S deposition was used in the present study, comprising both dry and wet deposition. Each type of deposition was modelled using regression kriging using EMEP precipitation as a linear regression model predictor, with residuals fitted using a linear variogram model with no nugget. Using the variogram model and linear model, deposition flux over the catchment area was predicted using block kriging with the target geometry being the catchment polygon. Missing values from 1985-1990 and 1990-1995 where estimated within each catchment using loess regression.

C) Extended output and additional analyses

Data standardization and software

All pre-processing and analyses was done in R v. 3.2.1 (*R Core Team 2015*). Prior to the analyses, TOC where log transformed, and the data where standardized by

detracting mean and dividing on standard deviation using the scale function of the *base* library.

We first tested for overall temporal trends in the time series of lake TOC and each of the catchment drivers using a regional Kendall trend test using the rkt library (*Marchetto 2015, rkt: Mann-Kendall Test, Seasonal and Regional Kendall Tests*). Each individual lake was used as block (*Helsel and Frans 2006, Env. Sci. Tech. 40:4066-4073*).

To test for effects of vegetation development (NDVI) after correcting for other known drivers of TOC, we included effects of S deposition, catchment NDVI, runoff, and air temperature as driver variables in a linear mixed effect model with lake TOC as dependent variable using the nlme library (*Pinheiro et al. 2012, nlme: Linear and Nonlinear Mixed Effects Models. R package version 3.1-104*). Year was included in the full model as a controlling variable (*Freckleton 2002, J. Anim. Ecol. 71:542-545*). Selection of random structure where done on a full fixed model structure (fitted by REML) by model comparison (*Zuur et al. 2009, Mixed effects models and extensions in ecology with R. Springer, New York*). This resulted in a full model including lake-ID as random intercept and year nested within lake-ID as random slope (???AIC > -76.65 in favor of the selected random structure). By introducing lake ID as random factor, we model between-lake variation in TOC caused by catchment variables not considered in the current study. For example static land cover properties that not are expected to change on the investigated time frame such as bogs and wetlands, catchment size, lake size. Temporal autocorrelation (AR1) was initially also tried, by entering year nested within lake-ID, but proved redundant in the initial selection of the random structure. There was no signs of multicollinearity (variance inflation factors, all VIF < 1.96, (Zuur et al. 2009) and maximum correlation between predictors where -0.61.

Catchment vegetation is expected to influence lake TOC with time lags (see main text). Due to catchment-specific properties such as fractions of alpine areas, forests and bogs, as well as inter-annual variability in climate and runoff, the time-lag between terrestrially fixed C (inferred as NDVI) and export of TOC may vary substantially. Based on visual inspection of cross-correlation plots between the dependent variable (TOC) and time lags of the explanatory variables, it was apparent that NDVI and runoff affected lake TOC with a considerable time lag. In comparison, there were no clear time-lags in the effect of S deposition or temperature.

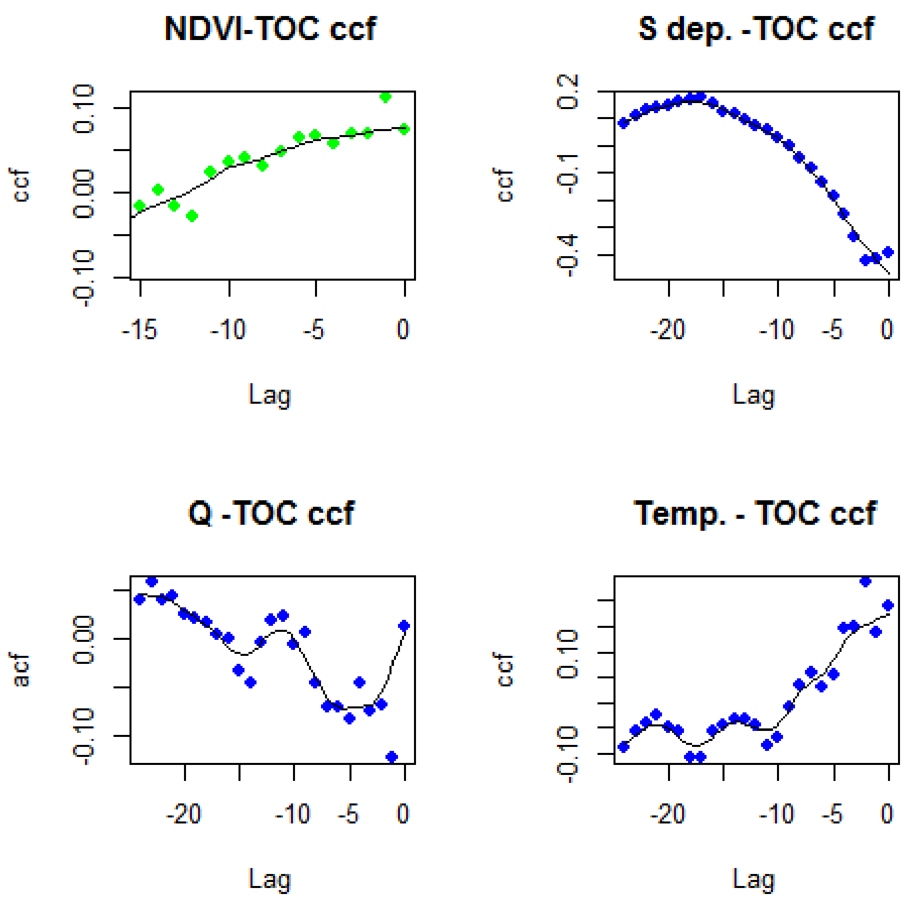

Figure S1: Inspecting for delayed response (time-lags) between drivers and response Here, we do a visual inspection of cross-correlation plots between the dependent variable (TOC) and time lags of the explanatory variables. The correlations are given as means across all lakes, and solid line represent a loess fitted curve for illustrational purposes.

NDVI and runoff affected lake TOC a considerable time lag. In comparison, there were no clear time-lags in the effect of Sulphur deposition or temperature. In order to avoid data-dredging issues, we selected *a priori* a one year time lag for NDVI and runoff as the input for our analyses. We subsequently performed sensitivity analyses on the effect of time-lag chosen by re-running the final model with different time-lags (see below). The main predictions were mainly insensitive to choice of time-lag, and hence only 1 year time lags are presented in the results of the main text. S deposition and temperature where entered without time lags.

Model selection and multi-model inference

Model selection and model averaging on fixed effect structure where done by model comparison using the MuMIn library (*Grueber et al. 2011, J. Evol. Biol. 24:699-711*). We compared all possible combinations of fixed factors and ranked candidate

models according to the Akaike information criterion (AIC), and also calculated

delta AIC (Δi) in relation to the highest ranked model, as well the Akaike Weigths (wi). There was no clear top-ranked candidate model, and we applied Akaike weigth-based averaging over the 95% confidence model set (cumulative AIC

weights of models $\geq$ 0.95) for estimating coefficients for the candidate models as well as their 95% confidence intervals. The relative influence (RI) of each variable was given as the summation of wi across all models including that variable in the 95% confidence model set (*Johnson and Omland 2004, Trend. Ecol. Evol. 19:101-108*).

Residuals of the final selected models were visually inspected for deviations from normality, heteroscedasticity, and spatial or temporal autocorrelation without finding evidence for violation of model assumptions (see below).

To quantify the relative strength of the different catchment drivers of TOC, we included effects of S deposition, catchment NDVI, runoff, and air temperature as driver variables in a linear mixed effect model with lake TOC as dependent variable (nlme library; Pinheiro et al. 2012). Year, was included in the full model as a controlling variable (sensu Freckleton 2002). There was no signs of multicollinearity (variance inflation factors, all VIF < 1.96, (Zuur et al. 2009) and maximum correlation between predictors where 0.61 (see figure below).

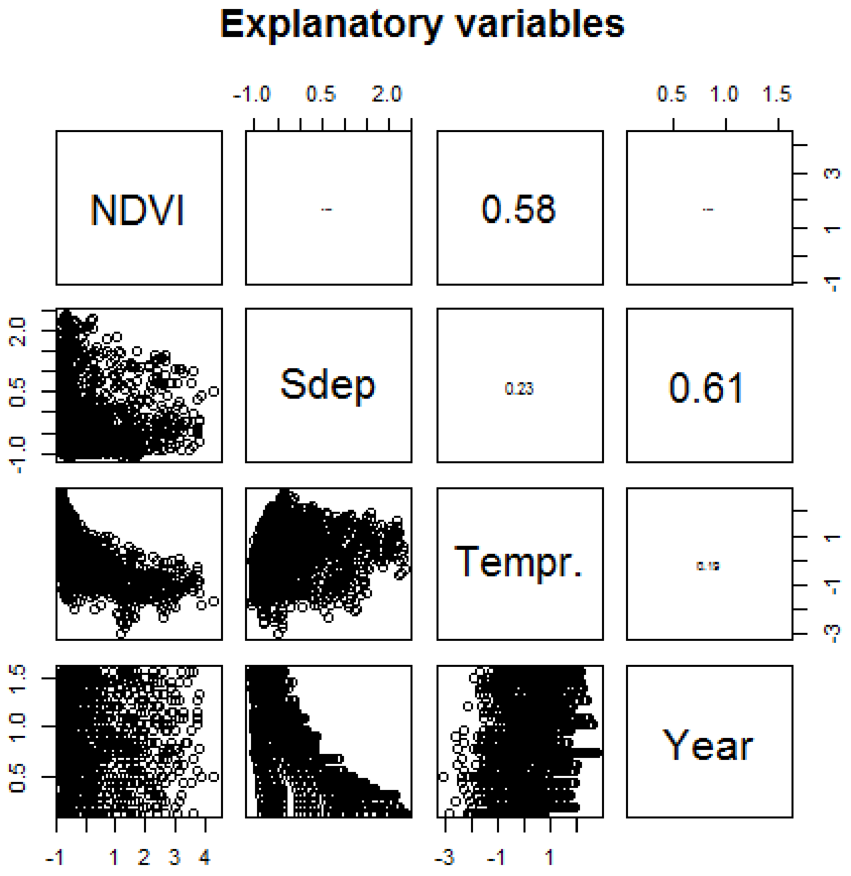

Figure S2:Scatter plot matrix of explanatory variables included in the analyses.

Analyses (R-code), for results as apparing in main text:

```
# comparing random structure
library(nlme)
library(MuMIn)
f1 <- formula(lnTOC ~ ndvi.summer_lag1 + q.summer_lag1 + sdep_pred +
tm.summer+year)

m1 <- gls(f1, data = data.std2, na.action = na.fail,method="REML")
m2 <- lme(f1, data = data.std2, random= ~1 |
as.factor(vatn_lnr),na.action = na.fail,method="REML")
m3 <- lme(f1, data = data.std2, random= ~year |
as.factor(vatn_lnr),na.action = na.fail,method="REML")

m4 <- lme(f1, data = data.std2, random= ~ 1 | as.factor(vatn_lnr),
          corAR1(form = ~ year |
```

```
as.factor(vatn_lnr)),method="REML",na.action = na.fail)
m5 <- lme(f1, data = data.std2, random= ~ year | as.factor(vatn_lnr),
          corAR1(form = ~ year |
as.factor(vatn_lnr)),method="REML",na.action = na.fail)

#Choosing random structure
AIC(m1,m2,m3,m4,m5)

# refitt model and model selection
m1 <- lme(f1, data = data.std2, random= ~ year |
as.factor(vatn_lnr),method="ML",na.action = na.fail)

dregde_mod <- dredge(m1,rank="AIC")

summary(m1)

avgmod.95p <- model.avg(dregde_mod, cumsum(weight) <= .95,fit=T)
dregdetable <- subset(dregde_mod, cumsum(weight) <= .95)
```

Table S2: Model selection tables of TOC against NDVI, runoff (Q), S deposition, temperature and year. The tables show parameter estimates for model terms included in the models, log likelihood (LogLik), AIC, AIC difference from best model (delta), and Akaike weigths (weigths). Only models from the top 95% confidence model set shown (cumulative AIC weight of models ≥ 0.95).

	Intercept	NDVI	Q	S dep	Tempr	year	df	logLik	AIC	delta	weight
24	-0.15	0.05	-0.06	-0.1	NA	0.15	9	-189.69	397.39	0.00	0.63
32	-0.15	0.05	-0.06	-0.1	-0.01	0.15	10	-189.56	399.11	1.72	0.26
23	-0.15	NA	-0.07	-0.1	NA	0.14	8	-192.94	401.89	4.50	0.07
22	-0.15	0.06	NA	-0.1	NA	0.15	8	-193.38	402.77	5.38	0.04

Table S3: Summary result for model averaging of fixed effects from the 95% confidence model set (cumulative Wi ≥ 0.95) of of TOC against NDVI, runoff (Q), S deposition, temperature and year.

	Estimate	Sd.Error	adj.SE	-95%CI	+95%CI
Intercept	-0.15	0.11	0.11	-0.36	0.06
NDVI	0.05	0.02	0.02	0.01	0.08
Q	-0.06	0.02	0.02	-0.10	-0.02
S dep.	-0.10	0.02	0.02	-0.14	-0.05
year	0.15	0.04	0.04	0.07	0.22
Tempr	-0.01	0.02	0.02	-0.04	0.02

Checking residual structure of final model

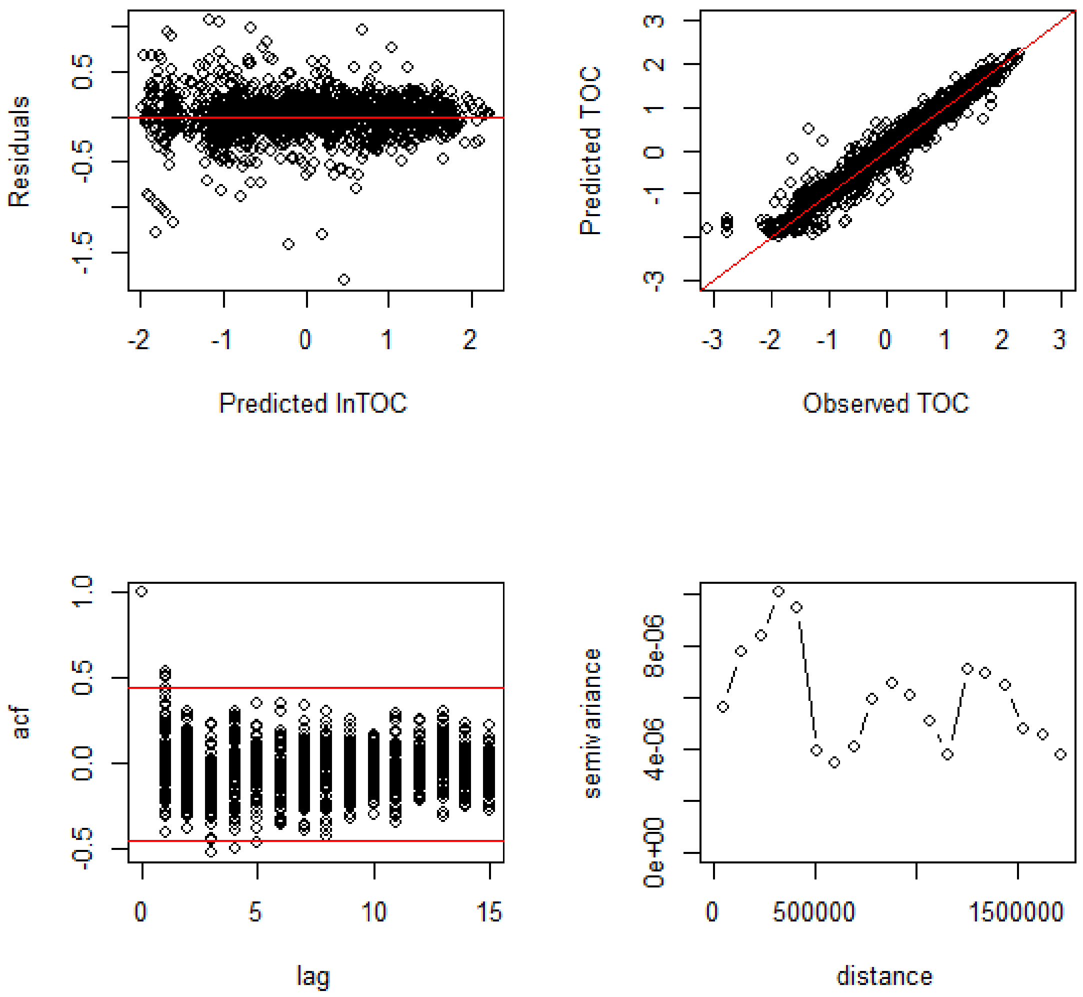

Figure S3: Residual plot of final model. Plotting predicted values for lnTOC against residuals from averaged modell (upper left pannel), observed against predicted lnTOC (upper rigth pannel), autocorrelation function (acf) for residuals (lower left pannel) and semivariogram for residuals (lower rigth pannel). Values are on standarized scale.Note pattern in residuals with a cluster of values in the lower end of the TOC scale (upper rigth pannels). Those are adressed below.

Re-running model without year as controlling variable

```
# comparing random structure
library(nlme)
library(MuMIn)
f1 <- formula(lnTOC ~ ndvi.summer_lag1 + q.summer_lag1 + sdep_pred +
tm.summer)

m1 <- gls(f1, data = data.std2, na.action = na.fail,method="REML")
m2 <- lme(f1, data = data.std2, random= ~1 |
as.factor(vatn_lnr),na.action = na.fail,method="REML")
m3 <- lme(f1, data = data.std2, random= ~ 1 | as.factor(vatn_lnr),
          corAR1(form = ~ year |
as.factor(vatn_lnr)),method="REML",na.action = na.fail)


#Choosing random structure
AIC(m1,m2,m3)

# refitt model and model selection
m1 <- lme(f1, data = data.std2, random= ~ 1 |
as.factor(vatn_lnr),method="ML",na.action = na.fail)

dregde_mod <- dredge(m1,rank="AIC")

summary(m1)

avgmod.95p <- model.avg(dregde_mod, cumsum(weight) <= .95,fit=T)
dregdetable <- subset(dregde_mod, cumsum(weight) <= .95)
```

Table S4: Model selection tables of TOC against NDVI, runoff (Q), S deposition, temperature. The tables show parameter estimates for model terms included in the models, log likelihood (LogLik), AIC, AIC difference from best model (delta), and Akaike weigths (weigths). Only models from the top 95% confidence model set shown (cumulative AIC weight of models $\geq$ 0.95).

	Intercept	NDVI	Q	S dep	Tempr	df	logLik	AIC	delta	weight
8	-0.05	0.05	-0.04	-0.22	NA	6	-235.38	482.75	0.00	0.52
6	-0.05	0.06	NA	-0.22	NA	5	-237.32	484.63	1.88	0.20
16	-0.05	0.05	-0.04	-0.22	0.00	7	-235.36	484.72	1.97	0.19
14	-0.05	0.06	NA	-0.22	0.01	6	-237.21	486.43	3.67	0.08

Table S5: Summary result for model averaging of fixed effects from the 95% confidence model set (cumulative Wi ≥ 0.95) of of TOC against NDVI, runoff (Q), S deposition, temperature.

	Estimate	Sd.Error	adj.SE	-95%CI	+95%CI
Intercept	-0.05	0.11	0.11	-0.27	0.17
NDVI	0.05	0.02	0.02	0.02	0.09
Q	-0.04	0.02	0.02	-0.08	0.00
S dep.	-0.22	0.01	0.01	-0.24	-0.20
Tempr	0.00	0.02	0.02	-0.03	0.03

Rerunning model exluding outliers

Outliers identified in residual plots (see above, standarized lnTOC < -2.5). The outliers are clusters of low TOC values. We have no knowlegde about potential causes for the outliers, and hence no a priori reason for exluding them from the analyses. One potential hypoteshis is, however, that this results from TOC beeing close to the detection limits of the instruments. We consecvently rerun the model selection process without these datapoints to check for influence. Although the relative importance of NDVI became sligtly lower, the main results of the model selection is similar, albeith a sligthly lower relative importance of NDVI. Hence, our conclution is that these outliers don't have any significant effect of our overall conclutions.

Analyses (R-code):

```
library(nlme)
library(MuMIn)

f1 <- formula(lnTOC ~ ndvi.summer_lag1 + q.summer_lag1 + sdep_pred +
tm.summer+year)

# re-running model exluding outliers
m1subset <- lme(f1, data = data.std2, random= ~ year |
as.factor(vatn_lnr),method="ML",na.action = na.fail,subset=lnTOC>-2.5)

dregde_mod_subset <- dredge(m1subset,rank="AIC")
avgmod.95p.subset <- model.avg(dregde_mod_subset, cumsum(weight) <=
.95,fit=T)
dregdetable <- subset(dregde_mod_subset, cumsum(weight) <= .95)
```

Table S6: Model selection tables model re-run without outliers. (TOC against NDVI, runoff (Q), S deposition, temperature and year). The tables show parameter

estimates for model terms included in the models, log likelihood (LogLik), AIC, AIC difference from best model (delta), and Akaike weigths (weigths). Only models from the top 95% confidence model set shown (cumulative AIC weight of models ≥ 0.95).

	Intercept	NDVI	Q	S dep	Tempr	year	df	logLik	AIC	delta	weight
24	-0.14	0.03	-0.06	-0.09	NA	0.15	9	-80.93	179.86	0.00	0.50
32	-0.14	0.03	-0.06	-0.09	-0.01	0.15	10	-80.56	181.12	1.25	0.27
23	-0.14	NA	-0.06	-0.09	NA	0.14	8	-82.68	181.36	1.50	0.24

Table S7: Summary result for model averaging of (model re-run without outliers) fixed effects from the 95% confidence model set (cumulative Wi ≥ 0.95) of of TOC against NDVI, runoff (Q), S deposition, temperature and year.

	Estimate	Sd.Error	adj.SE	-95%CI	+95%CI
Intercept	-0.14	0.10	0.10	-0.34	0.06
NDVI	0.03	0.02	0.02	0.00	0.06
Q	-0.06	0.02	0.02	-0.10	-0.02
S dep.	-0.09	0.02	0.02	-0.13	-0.05
year	0.15	0.04	0.04	0.07	0.22
Tempr	-0.01	0.01	0.01	-0.04	0.02

Re-running model selection with different time lags for NDVI and runoff(Q)

We performed sensitivity analyses on the effect of chosing of time-lag by re-running the final model with different time-lags. Model selection was run through all combinations of NDVI and runoff(Q) lags from 0 to 5, and the relative importance of NDVI in the model recorded. The main predictions were mainly insensitive to choice of time-lag, and hence only 1 year time lags are presented in the results of the main text. S deposition and temperature where entered without time lags.

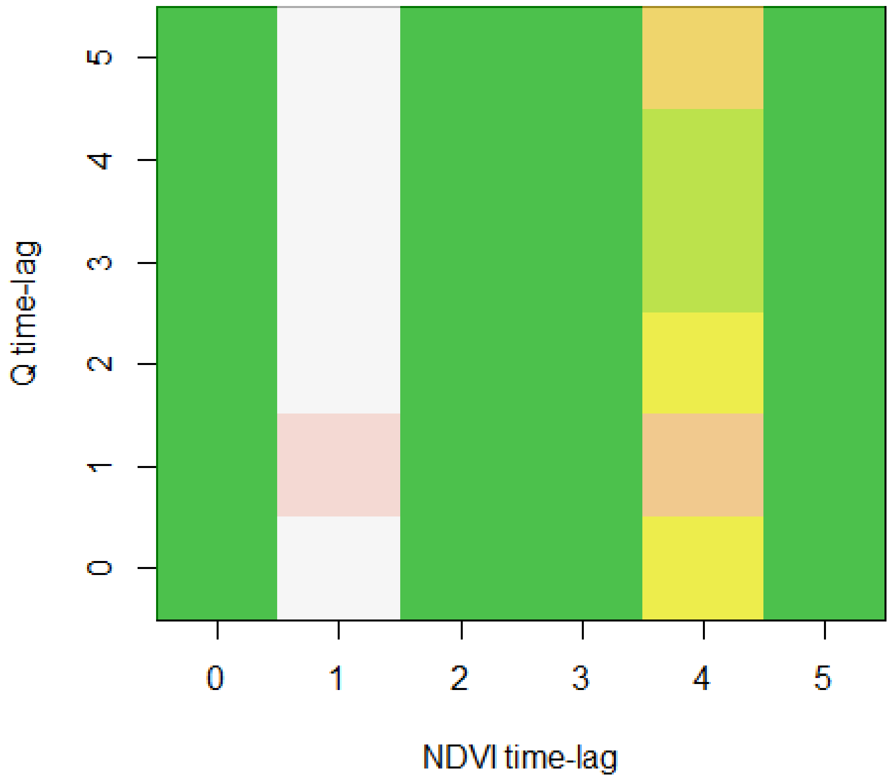

Figure S4: Visual presentation of support for NDVI effect using different time lags for NDVI and runoff. The effect of different time-lags on the relative importance of NDVI visualized by plotting the relative importance of NDVI along different time lags of runoff (Q) and NDVI. Range in relative importance is from 0.22 (dark green) to 0.99 (red). All time-lag combinations investigated included NDVI in the top ranked models (delta AIC < 2).

Substituting S deposition with SO4

We then illustrate the effect of substituting SO4 with NDVI in the original model. No major differences on model selection results by subtituting S deposition with SO4 measured in water. NDVI had for all practical purposes the same importance as in the orignal model using S deposition model. Hence, the two explanatory variables (S deposition and SO4) yielded comparable results with respect to model output.

Analyses (R-code):

```
library(nlme)
library(MuMIn)

f1 <- formula(lnTOC ~ ndvi.summer_lag1 + q.summer_lag1 + SO4 +
```

```
tm.summer+year)

m1subset <- lme(f1, data = data.std2, random= ~ year |
as.factor(vatn_lnr),
          method="ML",na.action = na.fail)

dregde_mod_subset <- dredge(m1subset,rank="AIC")
avgmod.95p.subset <- model.avg(dregde_mod_subset, cumsum(weight) <=
.95,fit=T)
dregdetable <- subset(dregde_mod_subset, cumsum(weight) <= .95)
```

Table S8: Model selection tables model on re-run substituting S deposition with SO4. (TOC against NDVI, runoff (Q), SO4, temperature and year). The tables show parameter estimates for model terms included in the models, log likelihood (LogLik), AIC, AIC difference from best model (delta), and Akaike weigths (weigths). Only models from the top 95% confidence model set shown (cumulative AIC weight of models ≥ 0.95).

	Intercept	NDVI	Q	S dep	Tempr	year	df	logLik	AIC	delta	weight
24	-0.07	0.05	-0.06	-0.20	NA	0.08	9	-169.78	357.56	0.00	0.55
32	-0.07	0.05	-0.06	-0.20	0.00	0.08	10	-169.78	359.56	2.00	0.20
8	0.02	0.05	-0.06	-0.24	NA	NA	8	-172.15	360.30	2.74	0.14
16	0.02	0.05	-0.06	-0.24	0.01	NA	9	-172.08	362.16	4.61	0.06
23	-0.07	NA	-0.07	-0.20	NA	0.08	8	-173.26	362.51	4.96	0.05

Table S9: Summary result for model averaging of (model re-run with S deposition substituted with SO4) fixed effects from the 95% confidence model set (cumulative Wi ≥ 0.95) of of TOC against NDVI, runoff (Q), SO4, temperature and year.

	Estimate	Sd.Error	adj.SE	-95%CI	+95%CI
Intercept	-0.15	0.11	0.11	-0.36	0.07
Q	-0.05	0.02	0.02	-0.09	-0.01
SO4	-0.11	0.02	0.02	-0.15	-0.06
year	0.14	0.04	0.04	0.07	0.21
NDVI	0.01	0.02	0.02	-0.02	0.05

D) Trend analyses

In this part of the supplementary, we investigated for the relative correspondence between the temporal trends in lake TOC and catchment drivers by using the Theil-Sen's slopes for lake TOC as dependent variable and slopes for mean catchment specific NDVI, runoff, atmospheric S deposition as explanatory variables using generalized least-squares (gls). We also tested for the inclusion of spatial autocorrelation structure comparing models with and without (fitted by REML) using Akaike's information criterion (AIC) (Zuur et al. 2009). However, no support for inclusion of a spatial autocorrelation was apparent (ΔAIC > 4.00 in support of model without spatial autocorrelation). The the tests is also repeated using relative trends (% change), as well as absolute change for sensitivity puroposes. We additionally present analyses where S deposition where substituted with SO4 as dependent variable, and finally re-run with trends expressed as percent change instead of slopes.

Trends (Theil-Sen's slopes) and S deposition as predictor

Table S10: Model selection tables for slopes of TOC against NDVI, runoff (Q) S dep. and temperature. The tables show parameter estimates for model terms included in the models, log likelihood (LogLik), AIC, AIC difference from best model (delta), and Akaike weigths (weigths). Only models from the top 95% confidence model set shown (cumulative AIC weight of models ≥ 0.95).

	Intercept	NDVI	Q	S dep	Tempr	df	logLik	AIC	delta	weigth
5	0	NA	NA	-0.26	NA	3	232.66	-459.33	0.00	0.35
6	0	0.08	NA	-0.27	NA	4	233.12	-458.23	1.09	0.20
7	0	NA	-0.20	-0.26	NA	4	232.87	-457.74	1.58	0.16
13	0	NA	NA	-0.26	0.18	4	232.72	-457.44	1.88	0.14
8	0	0.08	-0.15	-0.27	NA	5	233.23	-456.47	2.86	0.08
14	0	0.08	NA	-0.27	0.10	5	233.13	-456.27	3.06	0.08

Table S11: Model selection tables for slopes of TOC against NDVI, runoff (Q) S dep. and temperature. The tables show parameter estimates for model terms included in the models, log likelihood (LogLik), AIC, AIC difference from best model (delta), and Akaike weigths (weigths). Only models from the top 95% confidence model set shown (cumulative AIC weight of models ≥ 0.95).

	Estimate	Sd.Error	adj.SE	-95%CI	+95%CI
Intercept	0.00	0.00	0.00	-0.01	0.00
S dep.	-0.26	0.03	0.03	-0.32	-0.21
NDVI	0.08	0.09	0.09	-0.10	0.26
Q	-0.19	0.32	0.33	-0.83	0.46
Tempr	0.15	0.55	0.56	-0.94	1.25

Using trends (Theil-Sen's slopes) but with measured SO4 in lake water samples as predictor

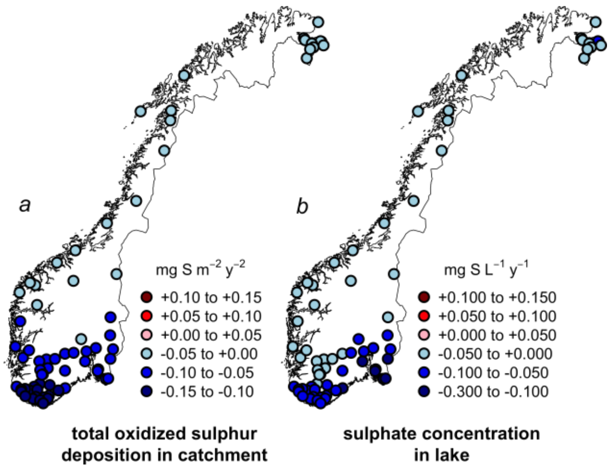

Figure S5: Geographical distribution of site-specific Theil-Sen's slope of total atmospheric oxidized sulphate deposition (catchment) and SO4-S concentration (lake). Figure created in R v. 3.2.1 (*R Core Team, 2015;* URL http://www.R-project.org/) using the libraries raster (*Hijmans, 2015*) and sp (*Bivand et al. 2013, Applied spatial data analyses with R Springer, NY*).

Table S12: Model selection tables for slopes of TOC against NDVI, runoff (Q), SO4 and temperature. The tables show parameter estimates for model terms included in the models, log likelihood (LogLik), AIC, AIC difference from best model (delta), and Akaike weigths (weigths). Only models from the top 95% confidence model set shown (cumulative AIC weight of models ≥ 0.95).

	Intercept	NDVI	Q	SO4	Tempr	df	logLik	AIC	delta	weigth
5	0	NA	NA	-0.2	NA	5	231.41	-452.82	0.00	0.38
13	0	NA	NA	-0.2	0.37	6	231.62	-451.23	1.59	0.17
6	0	-0.04	NA	-0.2	NA	6	231.48	-450.96	1.86	0.15
7	0	NA	-0.10	-0.2	NA	6	231.47	-450.93	1.89	0.15
14	0	-0.06	NA	-0.2	0.45	7	231.78	-449.55	3.27	0.07
15	0	NA	-0.09	-0.2	0.35	7	231.66	-449.32	3.50	0.07

Table S13: Summary result for model averaging of fixed effects from the 95% confidence model set (cumulative Wi ≥ 0.95) of slopes of TOC against NDVI, runoff (Q), SO4 and temperature.

	Estimate	Sd.Error	adj.SE	-95%CI	+95%CI
Intercept	0.00	0.01	0.01	-0.01	0.01
SO4.	-0.20	0.04	0.05	-0.29	-0.11
NDVI	0.39	0.58	0.59	-0.77	1.54
Q	-0.04	0.10	0.10	-0.24	0.16
Tempr	-0.10	0.32	0.32	-0.74	0.54

Using relative slopes (% change) with S deposition as driver

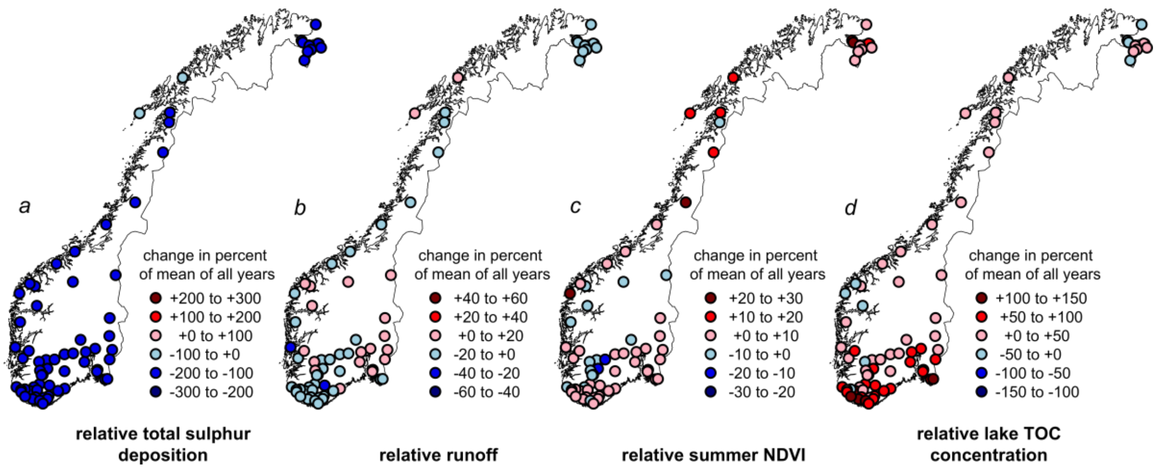

Figure S6: Site-specific percent change of S deposition, runoff, summer NDVI, and lake TOC concentration, relative to the mean of all years available at each lake and catchment. Figure created in R (*R Core Team, 2015*) R v. 3.2.1 (*R Core Team, 2015;* URL http://www.R-project.org/) using the libraries raster (*Hijmans, 2015*) and sp (*Bivand et al. 2013, Applied spatial data analyses with R Springer, NY*).

Table S14: Model selection tables for relative slopes (percent change) of TOC against NDVI, runoff (Q), S deposition and temperature. The tables show parameter estimates for model terms included in the models, log likelihood (LogLik), AIC, AIC difference from best model (delta), and Akaike weigths (weigths). Only models from the top 95% confidence model set shown (cumulative AIC weight of models ≥ 0.95).

	Intercept	NDVI	Q	S dep	Tempr	df	logLik	AIC	delta	weight
13	-61.79	NA	NA	-0.89	-4.77	4	-340.88	689.75	0.00	0.49
14	-76.80	0.5	NA	-0.97	-4.56	5	-340.57	691.14	1.39	0.24
15	-62.21	NA	-0.05	-0.89	-4.75	5	-340.87	691.74	1.99	0.18
16	-77.10	0.5	-0.04	-0.97	-4.55	6	-340.57	693.14	3.38	0.09

Table S15: Summary result for model averaging of fixed effects from the 95% confidence model set (cumulative Wi ≥ 0.95) of slopes (percent change) of TOC against NDVI, runoff (Q), S deposition and temperature.

	Estimate	Sd.Error	adj.SE	-95%CI	+95%CI
Intercept	-66.89	31.34	31.89	-129.39	-4.39
S dep.	-0.91	0.19	0.19	-1.29	-0.53
Tempr	-4.69	1.77	1.80	-8.22	-1.17
NDVI	0.50	0.66	0.67	-0.82	1.81
Q	-0.05	0.48	0.48	-1.00	0.90
